# Current status, perceptions, and barriers regarding weight loss approaches in China

**DOI:** 10.1007/s12020-025-04315-7

**Published:** 2025-07-01

**Authors:** Ziwei Lin, Hao Zhu, Si Si, Jiawei Xu, Esther Artime, Swarna Khare, Victoria Higgins, Andrea Leith, Yan Bi

**Affiliations:** 1 Eli Lilly and Company, Suzhou, China; 2 Eli Lilly and Company, Alcobendas, Spain; 3 Eli Lilly and Company, Bracknell, UK; 4 Adelphi Real World, Bollington, UK; 5 Nanjing Drum Tower Hospital affiliated to Nanjing University Medical School, Nanjing, China

**Keywords:** China, Cross-sectional, Obesity, Overweight, Real-world evidence, Weight control

## Abstract

**Purpose:**

To describe management and barriers regarding weight loss in China.

**Materials and methods:**

Data were from the Adelphi Real World Obesity Disease Specific Programme™, a cross-sectional survey between April and July 2022 in Chinese clinical practice. Physicians managing people with obesity or overweight (PwO) and PwO aged ≥ 18 years, under weight management programs, and/or with a body mass index ≥ 28 kg/m^2^ were eligible.

**Results:**

100 physicians and 801 PwO were included. Before current clinical management, PwO had attempted self-management using their own diet (87%) and exercise (84%) programs, natural remedies/diet pills sold on the internet (26%) or over the counter diet pills (19%). Physicians reported that typical first-line weight management methods in clinical practice were diet (82%) and exercise (82%), with half (50%) using drug approaches. Only 19% of PwO reported full compliance with diet and exercise programs. Among the 78 physicians who used anti-obesity medications (AOMs), the single most important reason for initiating AOM was failure to reach the desired weight loss with diet and exercise alone (58%). Of the 78 physicians, 67% felt that most weight loss approaches always fail and 73% felt that AOM options were restricted; the top rated single most desired improvements in future AOMs were improved or greater efficacy (26%) and safer long-term use (26%). Of PwO, most (88%) had some willingness to try a new AOM.

**Conclusion:**

PwO usually self-managed initial weight loss attempts. Physicians typically initiated lifestyle interventions as first-line treatment despite low PwO compliance. Both physicians and PwO desired improved AOMs. Of note, no approved prescription-only AOMs were available in China during the time window of the study.

## Introduction

The prevalence of obesity and overweight in China has risen rapidly over recent decades [1] and effective clinical weight management is therefore crucial [2]. However, physicians in China currently have limited options for weight management in people with obesity or overweight (PwO) [2]. Lifestyle interventions are the first-line treatment for obesity [2, 3], but the weight loss effect is modest, and weight regain is common after PwO discontinue the program [4]. If lifestyle interventions are unsuccessful, the addition of anti-obesity medications (AOMs) can be considered, but the availability of these agents in China is limited [2, 3]. Until the end of 2022, the only approved medication for weight loss management was orlistat, a gastrointestinal lipase inhibitor, which has limited efficacy in promoting weight reduction and is associated with adverse events that can lead to treatment discontinuation [5, 6]. During this time, agents approved for type 2 diabetes, such as glucagon-like peptide-1 (GLP-1) receptor agonists and metformin [2, 3], were also used off-label for weight reduction. Bariatric surgery can be conducted in people with severe obesity, but acceptance of surgical intervention is low due to the invasive nature and high costs of the procedure [2].

A previous analysis of data from the Adelphi Real World Obesity Disease Specific Programme^TM^ (DSP^TM^) showed that few PwO in China achieved their weight loss targets using available weight loss methods, and that both PwO and physicians were unsatisfied with the outcomes of weight loss attempts [7]. Moreover, the survey-based ACTION-China study identified that the most common weight loss methods selected by PwO and healthcare providers were diet and exercise, with only around one third of physicians and less than 3% of PwO considering prescription weight-loss medication (from a hospital) to be effective [8]. While previous data suggest limited use of AOMs in China, further insights into weight loss approaches are lacking. The aim of the present analysis was to describe current weight loss approaches in China and provide a comprehensive understanding of the perceptions, attitudes, barriers, and expectations regarding weight loss management, with a focus on AOMs, among Chinese physicians and PwO. Our findings may help to identify unmet needs and reveal opportunities to improve weight management in China.

## Materials and methods

### Study design and data sources

Data for this analysis were obtained from the Adelphi Real World Obesity DSP, a cross-sectional survey with retrospective data collection including physicians and PwO in their clinical practice in China between April and July 2022. The DSP methodology, initiated in 1995, provides a large multinational, cross-sectional survey with consistent methodology generating data from real-world clinical practice across multiple therapy areas, including obesity, and has been previously validated and described in detail [9–12]. DSPs are designed without a prior hypothesis to understand patients, disease impact and associated treatment/management approaches, and are adaptable to different countries, cultures, and disease areas. This analysis is a secondary data analysis from an existing secondary DSP database. Data reported here were captured from physician surveys, detailed physician-reported questionnaires, and patient-reported questionnaires. Only data available to the physician at the time of the consultation were collected, including information from conversations between the physician and PwO during consultations and retrospective data from medical records.

### Study population

Eligible physicians were diabetologists/endocrinologists and internists personally responsible for managing and treating PwO, who must see at least 16 PwO per month, and accept all survey requirements. To avoid selection bias, participating physicians were randomly chosen from public listings of Chinese healthcare physicians by independent fieldwork partners familiar with local healthcare systems. Physicians completed an online survey and were asked to complete a physician-reported online questionnaire for their next eight consecutive PwO attending their clinic who met the inclusion criteria. The number of PwO was chosen to maximize the number of physicians sampled while minimizing the burden on each physician.

PwO were eligible for inclusion if, at the time of data collection, they were aged ≥ 18 years, enrolled in a weight management program and/or had a body mass index ≥ 28 kg/m^2^ (based on Chinese criteria for obesity [2]). PwO receiving off-label metformin, GLP-1 receptor agonists or over the counter (OTC) AOMs were included. PwO participating in an obesity clinical trial at the time of data collection were not eligible to be included in the DSP. The same PwO for whom physicians completed physician-reported questionnaires were asked to complete voluntary patient-reported questionnaires. PwO completed questionnaires independently of their physician immediately after the consultation and returned them in a sealed envelope to ensure confidentiality.

During the time window of this survey, no approved prescription-only AOMs were available in China; orlistat was the only medication approved for weight loss management and was available OTC.

### Outcome measures

Physicians completed an online survey in which they were required to indicate their medical specialty and recall the overall trends for obesity, PwO seeking health care, current clinical practice for weight management, and current and preferred AOM profiles based on their clinical experience in the past 12 months. Using data previously collected during routine consultations, physicians also provided demographic and medical information for participating PwO, as well as their evaluations and expectations for the participant’s weight loss experience to date, using a physician-reported questionnaire. Using a patient-reported questionnaire, PwO self-reported on a voluntary basis their weight loss targets and experiences of past weight loss attempts, including number of attempts, weight loss experience and reasons for discontinuing attempts at weight loss.

### Statistical analysis

All analyzes were descriptive. Categorical variables were reported using frequencies and percentages. Continuous variables were reported as mean and standard deviation (SD) or median and interquartile range (Q1, Q3). Characteristics of the participating physicians and PwO, current weight loss approaches according to physicians and PwO, perceptions and use of AOMs according to physicians, and PwO’s perspectives on AOMs were described. In the questionnaires, the term AOM included OTC AOMs (orlistat), medicines used off-label for weight loss (metformin and GLP-1 receptor agonists), and natural remedies.

## Results

### Study population

Overall, 100 physicians and 801 PwO in China were included. The characteristics of physicians and PwO have been reported previously and are described here in brief [7]. Of the 100 participating physicians, 50 specialized in endocrinology and 50 in internal medicine. Among the 801 participating PwO, the mean (SD) age was 38.3 (11.7) years and 55% were female. At diagnosis and after a median (Q1, Q3) of 6.4 (3.9, 12.1) months following a weight loss program under the guidance of a physician during the current weight loss attempt, respectively, the mean (SD) body mass index (BMI) was 32.3 (4.5) kg/m^2^ and 30.9 (4.3) kg/m^2^. Of the 801 PwO, 795 filled out patient-reported questionnaires.

### Weight loss approaches and treatment patterns in China

**Fig. 1:**
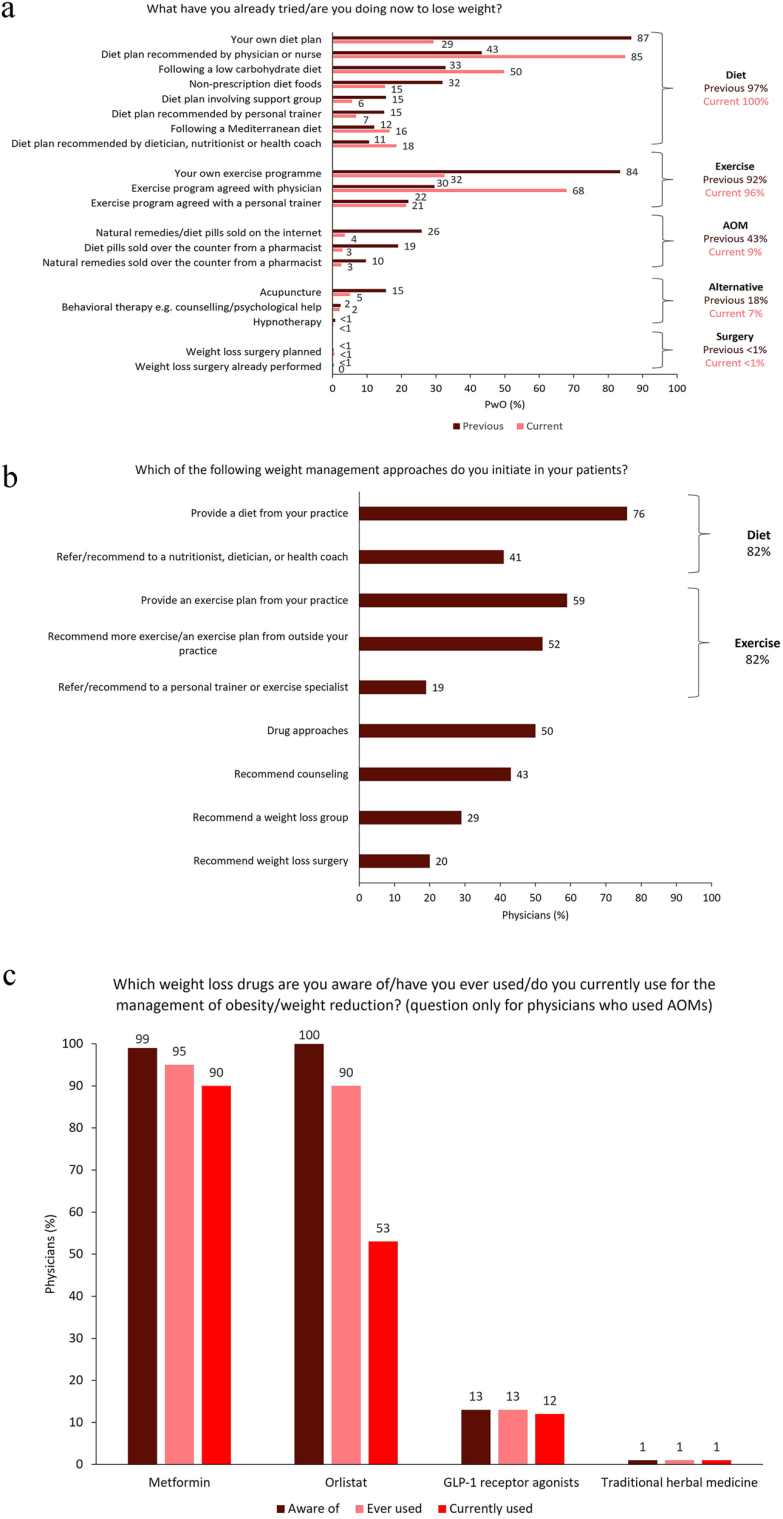
(**a**) Previous and current weight management approaches reported by PwO (*N* = 795); (**b**) Weight management approaches initiated by physicians in PwO (*N* = 100); (**c**) AOMs currently used for weight management among physicians who use AOMs (*N* = 78). AOM anti-obesity medication, GLP-1 glucagon-like peptide-1, PwO people with obesity or overweight

Lifestyle interventions were the most common weight management approaches according to PwO (Fig. 1a) and physicians (Fig. 1b). Prior to current clinical management, most PwO reported that they had attempted to lose weight using diet (97%) and exercise (92%), mainly via self-management using their own diet plan (87%) and exercise program (84%) (Fig. 1a). In addition, 43% of PwO had previously taken AOM that had not been prescribed by a physician, including natural remedies/diet pills sold on the internet (26%) or OTC diet pills from a pharmacist (19%). Under current clinical management, the most common approaches were also diet (100%) and exercise (96%), with most PwO following a diet plan recommended by their physician or nurse (85%) and an exercise program agreed upon with their physician (68%). Only 9% of PwO were currently taking AOM that had not been prescribed by a physician. Physicians reported that the most common approaches initiated for the weight management of PwO in clinical practice were diet (82%) and exercise (82%), with half (50%) initiating weight management using drug approaches (Fig. 1b).

Of the 100 physicians in the survey, 78 used AOMs and 22 had not used AOMs for weight management. Among the 78 physicians who used AOMs, metformin (off-label) was the most common AOM ever and currently used (95 and 90%, respectively) (Fig. 1c). Orlistat (OTC) had previously been used by 90% of physicians who used AOMs but was currently used by only 53%. During the time window of this study, GLP-1 receptor agonists were only approved for type 2 diabetes in China (since 2011), and awareness of these agents was low among physicians who used AOMs (13%). A small proportion of physicians (13%) had previously used GLP-1 receptor agonists for weight management, and most had continued to use these agents (12%) (Fig. 1c).

### Physician perspectives on AOMs

Table 1 details the perspectives on the use of AOMs that were available at the time of data collection among the 78 physicians who used AOMs. The single most important reason for initiating AOM was if the desired weight loss had not been achieved with diet and exercise alone (58%), followed by poor PwO compliance with the diet and exercise program (36%). Most physicians who used AOMs (71 and 60%, respectively) agreed that there was a minimum BMI level (mean 29.2 and 29.3 kg/m^2^, respectively) that needed to be reached before initiating an AOM in PwO without or with obesity-related complications (ORCs). Nevertheless, around one third of physicians did not set a minimum BMI threshold for initiation of an AOM in PwO without or with ORCs (29 and 40%, respectively). More than half of physicians (59%) did not set a maximum duration for using AOMs, while 41% indicated that there was a maximum time period (mean 12.9 months) beyond which they considered it unsuitable to continuously use an AOM in individual PwO. The main reasons for withdrawing treatment with AOM were PwO complaints about drug side-effects (77%), achievement of weight loss goals (62%), and the AOM not having the desired effect on PwO’s weight loss (58%).

**Table 1:** Perspectives on AOM use among physicians who use AOMs (*N* = 78)

	*N*	Value
Single most important reason to use an AOM rather than rely on diet and exercise alone, *n* (%)	78	
Diet and exercise tried but did not produce desired weight loss		45 (58)
Poor PwO compliance with diet and exercise program		28 (36)
Diet and exercise successful but now need to maintain weight loss achieved		5 (6)
Is there a minimum BMI level that needs to be reached before considering AOM use?	78	
For PwO without ORCs		
Yes, *n* (%)		55 (71)
Minimum BMI, kg/m^2^, mean (SD)	43	29.2 (8.1)
For PwO with ORCs		
Yes, *n* (%)		47 (60)
Minimum BMI, kg/m^2^, mean (SD)	43	29.3 (6.6)
Is there a maximum time period beyond which you would consider it unsuitable to continuously use an AOM in individual PwO?	78	
Yes, *n* (%)		32 (41)
Maximum time period, months, mean (SD)	32	12.9 (6.8)
Under what circumstances, if any, would you withdraw an AOM, *n* (%)	78	
PwO complaint about drug side-effects		60 (77)
PwO achievement of weight loss goals		48 (62)
AOM not having desired effect on PwO’s weight loss		45 (58)
PwO need a break from AOM		42 (54)
Poor PwO adherence to AOM		38 (49)
Other PwO conditions (e.g. diabetes, cardiovascular concerns, mental health concerns) become the treatment priority		33 (42)
PwO have addressed lifestyle factors associated with weight gain		29 (37)
PwO failing to address lifestyle factors associated with weight gain		11 (14)

*AOM* anti-obesity medication, *BMI* body mass index, *ORC* obesity-related complication, *PwO* people with obesity or overweight, *SD* standard deviation

**Fig. 2:**
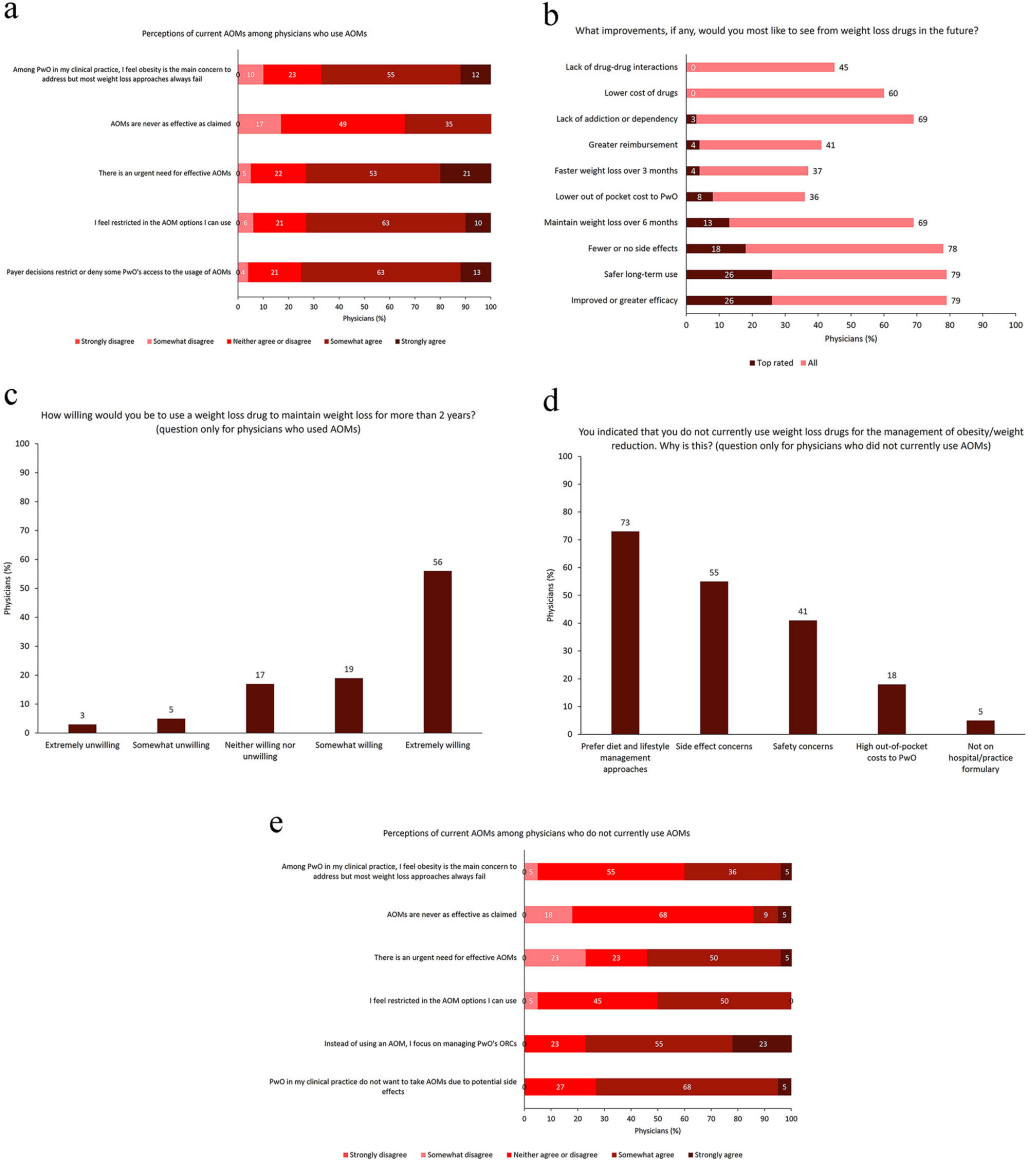
(**a**) Perceptions of current AOMs among physicians who use AOMs (*N* = 78); (**b**) Most desired future improvements in AOMs according to physicians who use AOMs (*N* = 78); (**c**) Willingness of physicians to use AOMs for more than 2 years among physicians who use AOMs (*N* = 78); (**d**) Reasons for not using AOMs among physicians who do not currently use AOMs (*N* = 22); (**e**) Perceptions of current AOMs among physicians who do not currently use AOMs (*N* = 22). AOM anti-obesity medication, ORC obesity-related complication, PwO people with obesity or overweight

The perceptions of AOMs available at the time of data collection and expectations for future AOMs among the 78 physicians who used AOMs are shown in Fig. 2a–c. Of these physicians, 67% somewhat or strongly agreed that obesity was the main concern to address in PwO but that most weight loss approaches always fail, 73% agreed that AOM options were restricted, and 74% agreed that there was an urgent need for effective AOMs (Fig. 2a). The top rated single most desired improvements in future AOMs were improved or greater efficacy (26%) and safer long-term use (26%) (Fig. 2b). Overall, 76% of physicians were willing to use AOMs for more than 2 years to maintain PwO’s weight loss, and 56% were extremely willing to do so (Fig. 2c). Of the 22 physicians who did not currently use AOMs, the main reasons for not doing so were preference for diet and lifestyle interventions (73%) and concerns over side effects (55%) and safety (41%) (Fig. 2d). Most physicians who did not use AOMs somewhat or strongly agreed their focus was on managing ORCs rather than using an AOM (77%) and that PwO do not want to take AOMs due to potential side effects (73%) (Fig. 2e). Even among physicians who did not use AOMs, more than half agreed that there was an urgent need for effective AOMs (Fig. 2e).

### PwO perspectives on AOMs

Only 19% of PwO reported that they fully complied with their diet and exercise programs (Table 2). Less than half of PwO (43%) had ever had an AOM suggested by their doctor, nurse or therapist and less than half (43%) had ever received an AOM. Among PwO who had ever received AOM, the main reason for taking an AOM was because they were worried about the effect of their weight on their health (65%). Relatively few PwO took an AOM based on medical advice regarding general health reasons (20%) or the presence of another condition which could worsen due to their weight (10%). Most PwO (88%) had some willingness to try a new AOM if it would reduce their weight (score 2–5 on a 5-point scale where 1 was ‘I do not like trying new medicine’ and 5 was ‘I am always ready to try new medicine’), while 44% had a strong willingness (score 4–5) (Table 2).

**Table 2:** Weight loss experiences in PwO (*N* = 795)

	*N*	*n* (%)
If you follow a diet and exercise program, which of these best describes how you feel about this?	795	
I follow it completely		155 (19)
I do my best to follow it but I sometimes fail		577 (73)
My diet and exercise program is not important. It is the medicine I am taking that will make me lose weight		63 (8)
Has your doctor, nurse, or therapist ever suggested that you try a medicine to help with your weight loss?	795	
Yes		342 (43)
No		434 (55)
Don’t know		19 (2)
Have you ever received an AOM?	795	
Yes		342 (43)
No		453 (57)
Thinking about your current and past AOM, what are or were the main reasons for you taking these (all answers that apply)?	342	
I was worried about the effect of my weight on my health		222 (65)
I want to improve my self-confidence around other people		151 (44)
My family/friends were concerned about my weight		127 (37)
I want to be more active		87 (25)
I heard and/or read about new treatments and weight-loss approaches		81 (24)
My doctor advised me for general health reasons		70 (20)
I want to live as long as I can		59 (17)
My doctor, nurse, or therapist advised me to because of another condition I have which could get worse due to my weight		35 (10)
How prepared are you to try new medicine if you think it will reduce your weight, on a 5-point scale where 1 was ‘I do not like trying new medicine’ and 5 was ‘I am always ready to try new medicine’?	795	
1		93 (12)
2		127 (16)
3		227 (29)
4		234 (29)
5		114 (14)

*AOM* anti-obesity medication, *PwO* people with obesity or overweight

## Discussion

Our findings showed that, in China, PwO seeking medical advice for weight loss tended to choose self-management before consulting with a physician. Physicians typically initiated lifestyle interventions as the first-line treatment for weight loss, yet only around one fifth of PwO reported full compliance with diet and exercise programs. AOMs were not widely used because of the limited choice of available agents and concerns regarding the safety of drug treatments. Physicians felt there was an urgent need for AOMs with improved efficacy and long-term safety profiles. In general, PwO were willing to try a new AOM if it would reduce their weight.

In the current analysis, we identified two major gaps in obesity management in China. Firstly, instead of seeking medical advice from the outset, PwO who wanted to lose weight tended to opt for self-management, with the most common weight loss methods being their own diet and exercise programs. This behavior may stem from two underlying factors. From one perspective, the ACTION-China study reported that 35% of PwO viewed weight loss as completely their own responsibility, and only 42% had discussed their excess weight and/or losing weight with a healthcare provider in the last 5 years [8]. Of the PwO who had discussed weight with a healthcare provider, the majority felt that the discussions were only a little (37%) or somewhat (43%) helpful [8]. Given that physicians typically recommend lifestyle interventions for weight loss, we speculate that PwO who have already tried and failed these approaches may view such advice as unhelpful and consequently be discouraged from consulting with a physician. From another perspective, physicians may fail to proactively initiate discussions about weight loss because they believe that PwO are not interested in losing weight or do not have the motivation to lose weight, and some may feel uncomfortable discussing weight unless PwO mention it first [13]. Together, the misperceptions from both sides may result in PwO choosing to self-manage their weight loss. However, using incorrect methods for weight management may lead to a cycle of weight loss and regain, as illustrated by a previous analysis showing that most PwO had a history of multiple weight loss attempts (mean of 6 attempts per person) [7]. Therefore, an urgent need exists for public education on weight management methods including encouragement of PwO to visit their physicians to discuss weight loss.

Secondly, physicians typically used lifestyle interventions rather than AOMs for weight loss management, with the single most important reason for initiating AOM being failure to reach the desired weight loss with diet and exercise alone. Furthermore, approximately one fifth of physicians never used AOMs primarily due to a preference for lifestyle interventions and concerns about the side effects and safety of the limited current options, and instead focused on the management of ORCs. As well as reflecting the limited AOM options at the time of data collection, these findings may reflect that physicians still do not regard obesity as a disease such as diabetes or hypertension for which they would certainly use medication, despite recognizing obesity as a precursor to those diseases [7]. Indeed, a previous analysis of data from the Adelphi Real World DSP showed that approximately 80% of physicians believed that obesity was caused by poor lifestyle choices [7]. These attitudes may partly explain why physicians primarily use lifestyle interventions to treat obesity. The clinical practice patterns observed in this analysis were consistent with current Chinese guidelines, which recommend that AOM is initiated for the treatment of obesity only after failure of first-line lifestyle interventions [2, 3].

However, first-line lifestyle interventions have limited efficacy and are inadequate for long-term weight management [2, 4]. In the present analysis, only around one fifth of PwO reported that they could fully comply with diet and exercise programs. Furthermore, the ACTION International Observation (ACTION-IO) study reported that only 31% of healthcare providers believed that PwO in their clinical practice were motivated to lose weight, and that in their opinion, only 35% of PwO had made a serious attempt to lose weight [13]. Our findings showed that most physicians, regardless of whether they currently used AOMs, agreed that current weight loss approaches always fail. Consistently, in a previous analysis using data from the Adelphi Real World Obesity DSP, most physicians (92%) and PwO (82%) were dissatisfied with the course of weight loss during the current weight loss attempt [7]. Indeed, the mean percentage weight loss was modest (4%), few PwO achieved the BMI target set by their physician or by themselves (10 and 2%, respectively), and more than half had failed to lose any weight or sustain any weight loss during the current attempt [7].

Another factor influencing the use of lifestyle interventions over pharmacotherapy is the limited availability of effective and safe AOMs in China, in contrast to the treatment of ORCs for which there are many established medications. During the time window of the current study, the only medication approved for weight management in China was orlistat (OTC) [2, 3], which has a modest weight loss effect in Chinese PwO at 24 weeks (imported orlistat: mean 4.5 kg [5.8%] of body weight) [6]. However, orlistat is associated with gastrointestinal adverse effects such as diarrhea, flatulence and fecal spotting, as well as the need for vitamin supplementation to prevent abnormalities in vitamin serum concentrations [14, 15]. In this analysis, almost half of physicians who had ever used orlistat did not currently use this medication, suggesting that they and the PwO in their clinical practice were unsatisfied with the treatment outcomes. The most frequently used AOM was metformin (off-label), which has a modest weight loss effect (mean 4.5 kg [5.1%] of body weight at 12 weeks) but a relatively favorable tolerability profile [16]. During the time window of this study, there were no GLP-1 receptor agonists approved for weight loss management in China [2, 3]. Nonetheless, 13% of physicians had used GLP-1 receptor agonists off-label, and 12% were currently using them, suggesting that healthcare providers tended to view these agents as efficacious and generally tolerable for weight loss management.

To address these challenges in the management of obesity, there is an urgent need to expand the armamentarium of available AOMs in China. Our findings showed that most PwO had at least some willingness (88%) or strong willingness (44%) to try new AOMs. Additionally, physicians felt there was an urgent need for improved AOMs in the future, with the most important attributes being safety for long-term use and improved or greater efficacy. The introduction of novel AOMs that meet these criteria may encourage physicians to prescribe medication for weight loss rather than relying on lifestyle interventions, as well as increasing the likelihood of PwO seeking medical help. Since the end of this study, two GLP-1 receptor agonists, liraglutide and beinaglutide, with a weight loss effect of 8% of body weight at 56 weeks (global data) [17] and 6% at 16 weeks [18], respectively, were approved for weight management in China in 2023. Two other agents with greater efficacy, namely semaglutide, a GLP-1 receptor agonist with a weight loss effect of 16.9% at 68 weeks (global data) [19], and tirzepatide, a GLP-1 and glucose-dependent insulinotropic polypeptide (GIP) receptor agonist with a weight loss effect of 22.5% at 72 weeks (15 mg; global data) [20], were approved for weight management in China in 2024. In addition, a phase 3 clinical trial of retatrutide, a GLP-1, GIP, and glucagon receptor agonist with a weight loss effect of 24.2% at 48 weeks (12 mg) in a phase 2 study [21], is ongoing in China. The potential future availability of improved AOMs will give physicians a wider range of options for the management of PwO.

The limitations of this study included the collection of data according to physicians’ recall and/or self-reported by PwO, such as the overall trends for obesity and information on PwO seeking health care in the past 12 months, which may be subject to recall bias and should be interpreted with caution. Recall bias, which may affect the responses of both physicians and patients, is a common limitation of surveys [10]. However, the data for these analyses were collected at the time of each patient’s appointment, which is expected to reduce the likelihood of recall bias. Moreover, few data on the use of GLP-1 receptor agonists were collected because their use was off-label during the time window of the current study [2, 3]. The generalizability of our findings is limited by the lack of availability of effective AOMs during data collection. While minimal inclusion criteria were used for selecting participating physicians, involvement may have been influenced by physicians’ willingness to complete the survey [10]. Identification of participating PwO was based on the physician’s judgement rather than a formalized diagnostic checklist but was representative of the physician’s real-world classification [10]. The DSP methodology does not require PwO to represent the general population in terms of race, income, social class or age [10]. Moreover, PwO without access to healthcare were not represented and the consecutive sampling may have caused over-representation of PwO who consulted their physician more frequently [10]. A further analysis exploring weight loss approaches by PwO’s characteristics would be of interest in the future.

In conclusion, our findings showed that PwO tended to self-manage their weight loss attempts, while physicians generally used lifestyle interventions rather than AOMs for weight loss management. Improved AOMs were desired by PwO and physicians. The expanding availability of more effective AOMs in China will provide physicians with a greater choice of weight management options and may encourage PwO to seek medical help. In March 2025, the National Health Commission of China launched the “Year of Weight Management” initiative, aimed at raising public awareness and improving skills in weight management through a 3-year systematic intervention. This policy is expected to significantly enhance the weight management landscape, with collaboration from all stakeholders, including healthcare providers, PwO, the general public, and the medical community.

## Data Availability

All data relevant to the analysis are included in the article. All data that support the findings of this survey are the intellectual property of Adelphi Real World. The datasets generated during and/or analysed during the current study are available upon reasonable request to Victoria Higgins at Victoria.Higgins@adelphigroup.com.
